# PACAP–PAC1 Signaling Regulates Serotonin 2A Receptor Internalization

**DOI:** 10.3389/fendo.2021.732456

**Published:** 2021-10-25

**Authors:** Atsuko Hayata-Takano, Yusuke Shintani, Keita Moriguchi, Naoki Encho, Kohei Kitagawa, Takanobu Nakazawa, Hitoshi Hashimoto

**Affiliations:** ^1^ Laboratory of Molecular Neuropharmacology, Graduate School of Pharmaceutical Sciences, Osaka University, Suita, Japan; ^2^ Molecular Research Center for Children’s Mental Development, United Graduate School of Child Development, Osaka University, Kanazawa University, Hamamatsu University School of Medicine, Chiba University and University of Fukui, Suita, Japan; ^3^ Department of Bioscience, Tokyo University of Agriculture, Setagaya-ku, Japan; ^4^ Division of Bioscience, Institute for Datability Science, Osaka University, Suita, Japan; ^5^ Transdimensional Life Imaging Division, Institute for Open and Transdisciplinary Research Initiatives, Osaka University, Suita, Japan; ^6^ Department of Molecular Pharmaceutical Science, Graduate School of Medicine, Osaka University, Suita, Japan

**Keywords:** pituitary adenylate cyclase-activating polypeptide (PACAP), internalization, hallucination, β-arrestin, G protein-coupled receptor (GPCR), serotonin 2A receptor (5-HT_2A_)

## Abstract

Mice lacking pituitary adenylate cyclase-activating polypeptide (PACAP) display psychomotor abnormalities, most of which are ameliorated by atypical antipsychotics with serotonin (5-HT) 2A receptor (5-HT_2A_) antagonism. Heterozygous *Pacap* mutant mice show a significantly higher hallucinogenic response than wild-type mice to a 5-HT_2A_ agonist. Endogenous PACAP may, therefore, affect 5-HT_2A_ signaling; however, the underlying neurobiological mechanism for this remains unclear. Here, we examined whether PACAP modulates 5-HT_2A_ signaling by addressing cellular protein localization. PACAP induced an increase in internalization of 5-HT_2A_ but not 5-HT_1A_, 5-HT_2C_, dopamine D_2_ receptors or metabotropic glutamate receptor 2 in HEK293T cells. This PACAP action was inhibited by protein kinase C inhibitors, β-arrestin2 silencing, the PACAP receptor PAC1 antagonist PACAP_6-38_, and PAC1 silencing. In addition, the levels of endogenous 5-HT_2A_ were decreased on the cell surface of primary cultured cortical neurons after PACAP stimulation and were increased in frontal cortex cell membranes of *Pacap^−/−^* mice. Finally, intracerebroventricular PACAP administration suppressed 5-HT_2A_ agonist-induced head twitch responses in mice. These results suggest that PACAP–PAC1 signaling increases 5-HT_2A_ internalization resulting in attenuation of 5-HT_2A_-mediated signaling, although further study is necessary to determine the relationship between behavioral abnormalities in *Pacap^−/−^* mice and PACAP-induced 5-HT_2A_ internalization.

## Introduction

Pituitary adenylate cyclase-activating polypeptide (PACAP) is a multifunctional neuropeptide that regulates a wide array of physiological responses, including emotion, cognition and motor function. It acts upon three G protein-coupled receptor subtypes: a PACAP-preferring receptor (PAC1) and two vasoactive intestinal polypeptide (VIP) receptors (VPAC1 and VPAC2) (1, 2). PAC1 signaling mediates cellular functions, such as transcriptional responses and cell survival, partly through its own internalization (3, 4). We previously reported that PACAP-deficient (*Pacap^−/−^*) mice show behavioral abnormalities such as locomotor hyperactivity in an open-field, deficits in prepulse inhibition (PPI) of the startle response, depression-like behavior and memory impairment (5–10). The hyperlocomotion and PPI deficits in *Pacap^−/−^* mice were reversed by risperidone, an atypical antipsychotic drug with antagonism of serotonin (5-HT)_2_ receptors and dopamine D_2_ receptors (D2) (10). The depression-like behavior in *Pacap^−/−^* mice were ameliorated by risperidone and the selective 5-HT 2A receptor (5-HT_2A_) antagonist, ritanserin (7). In addition, *Pacap^−/−^* mice (7) and heterozygous mutant mice (*Pacap* ^+^ *^/−^*) (11) show exaggerated (±)-2,5-dimethoxy-4-iodoamphetamine (DOI)-induced head-twitch responses compared with wild-type mice. *Pacap^−/−^* mice also have increased 5-HT content and 5-HT-immunoreactive cell counts in the dorsal raphe (12) and slightly decreased levels of the 5-HT metabolite, 5-hydroxyindoleacetic acid, in the cortex and striatum (5). These findings indicate that 5-HT_2A_ function may be involved in psychiatric conditions in which PACAP signaling is dysfunctional and that functional crosstalk may exist between PACAP and 5-HT_2A_ signaling pathways. However, the underlying molecular mechanisms for this remain unclear.

5-HT_2A_ has been implicated in many psychiatric disorders, such as schizophrenia and affective disorders (13). Clinical studies have indicated that impaired 5-HT_2A_ signaling plays a major role in schizophrenic episodes (14). Almost all currently available atypical antipsychotic drugs possess antagonistic effects against D2 and 5-HT_2A_ (15). Cellular internalization is known to play a critical role in the regulation of 5-HT_2A_ functions (16, 17). 5-HT, dopamine, DOI and clozapine induce 5-HT_2A_ internalization and recycling, and the signaling processes through which each ligand induces its effect are differentially regulated (17). In addition, different classes of G-protein-coupled receptors (GPCRs) can form heteromeric complexes that potentially contribute to the regulation of receptor internalization or alteration of pharmacological signaling properties (18, 19). 5-HT_2A_/metabotropic glutamate receptor 2 (mGlu2) and 5-HT_2A_/D2 form heteromeric complexes that induce unique hallucinogen-specific signaling (20–23). Thus, the signaling pathways involved in 5-HT_2A_ function are complicated, and the precise signaling pathways responsible for hallucinogenic and therapeutic effects remain unclear.

Our previous studies indicated that there are no significant differences in 5-HT content in the cortex and striatum or in 5-HT_2A_ protein levels in the somatosensory cortex between *PACAP* mutant and wild-type mice (5, 11, 24). Therefore, here, we examined the effect of PACAP signaling on 5-HT_2A_ internalization and revealed that the PACAP–PAC1 signaling pathway regulates 5-HT_2A_ internalization in a protein kinase C (PKC)- and β-arrestin2-dependent manner. These results further suggest the existence of functional crosstalk between PACAP and 5-HT_2A_-mediated signaling pathways in the brain.

## Materials and Methods

### Animals

ICR mice were purchased from Japan SLC (Shizuoka, Japan). Generation of *Pacap^−/−^* mice by gene targeting was reported previously (5). *Pacap^−/−^* mice and wild-type littermates on the ICR background were obtained by crossing *Pacap^+/−^* heterozygous mice.

All animal care and handling procedures were performed in accordance with protocols approved by the Animal Care and Use Committee of the Graduate School of Pharmaceutical Sciences, Osaka University. All efforts were made to minimize the number of animals used.

### Drugs

PACAP (PACAP-38, 4221-v), PACAP_6-38_ (4286-v) and VIP (4110-v) were purchased from Peptide Institute (Osaka, Japan). D-sphingosine (S7049), H89 (B1427) and 5-HT hydrochloride (H9523) were purchased from Sigma-Aldrich (St Louis, MO, USA). PD98059 (513000) was purchased from Calbiochem (CA, USA). H7 (BML-EI148) and HA1004 (BML-EI184) were purchased from ENZO Life Science (NY, USA).

### Vector Construction

The vector, pFN21A (HaloTag technology, Promega, Madison, WI, USA), encoding the secretory IL-6 signal peptide fused to the N-terminus of Halo-tag was a gift from Dr. Nagase (Kazusa DNA Research Institute). To generate the Halo-PAC1 construct, the hop1 splicing variant of a human *PAC1* cDNA was subcloned into the pFN21A vector at *Sgf*I and *Pme*I restriction sites as described previously (4). Human *5-HT_1A_*, *5-HT_2A_*, *D2* and *mGlu2* cDNAs were obtained from the Kazusa Collection of Flexi ORF Clones (Kazusa DNA Research Institute, Chiba, Japan). These clones were also subcloned into the pFN21A vector at *Sgf*I and *Pme*I restriction sites.

### Receptor Internalization in HEK293T Cells

Receptor internalization was quantitatively assessed using HaloTag technology (Promega) as described previously (4). HEK293T cells were maintained in Dulbecco’s modified Eagle’s medium (DMEM, 5919, Nissui, Tokyo, Japan) supplemented with 10% fetal bovine serum. The cells were transfected with Halo-expressing vector and labeled with the cell-impermeable Alexa Fluor 488 ligand (Promega) in Opti-MEM for 15 min at 37°C. Each inhibitor or antagonist pretreatment was for 30 min. The cells were then treated with 1 µM PACAP, 5-HT or saline, washed with phosphate-buffered saline and fixed in 4% paraformaldehyde. Cells were imaged using an FV1000D confocal microscope (Olympus, Tokyo, Japan) in sequential mode and membrane protein internalization was quantified using ImageJ software (NIH, MD, USA). To assess the internalization ratio, we defined the shape of a whole-cell (region of interest, ROI, A) and its cytoplasmic region (ROI B) by reducing the size by 5–10 pixels and then determining the fluorescence in both ROIs. The internalization ratio (%) was defined by dividing the amount of luminescence in ROI B by that in ROI A.

### β-Arrestin Silencing

siRNA-mediated silencing of β-arrestins was performed exactly as described in our previous study (4). β-arrestin1 (6218S; Cell Signaling Technology, Danvers, MA, USA), β-arrestin2 (sc-29743; Santa Cruz Biotechnology, Dallas, TX) or control siRNA (6568S; Cell Signaling Technology), each at 25 mM, were transfected using Lipofectamine RNAiMAX (Invitrogen) according to the manufacturer’s protocol. We confirmed that the β-arrestin1 and β-arrestin2 siRNAs effectively decreased the respective β-arrestin levels to less than 35% in HEK293T cells in our previous study (4).

### Antibodies

The following commercially available antibodies were used: rabbit polyclonal anti-PAC1 (ab54980, Abcam, Cambridge, UK), rabbit polyclonal anti-5-HT_1A_ (ab44635, Abcam), rabbit polyclonal anti-5-HT_2A_ (ab16028, Abcam), rabbit polyclonal anti-D2 (ab21218, Abcam), rabbit polyclonal anti-mGlu2/3 (06-676, Millipore, Darmstadt, Germany), mouse monoclonal anti-β-actin (MAB1501, Millipore), mouse monoclonal anti-alpha 1 sodium potassium ATPase (ab7671, Abcam). Horseradish peroxidase-conjugated anti-rabbit IgG and anti-mouse IgG were purchased from Cappel (Cochranville, PA, USA).

### Surface Biotinylation Assay and Membrane Protein Isolation

A receptor biotinylation assay was performed using the Pierce cell surface protein isolation kit (Thermo Fisher Scientific, Waltham, MA, USA) as described previously (25). Primary cultures of cortical neurons were prepared as described previously (4). The surface proteins of mouse primary cultured cortical neurons at 14 days *in vitro* were biotinylated with EZ-Link Sulfo-NHS-SS-biotin for 30 min at 4°C. To collect the surface proteins, cells were lysed with lysis buffer and biotinylated proteins were precipitated with NeutrAvidin agarose. The collected surface proteins were analyzed by western blotting.

Membrane protein isolation was performed using a plasma membrane protein isolation kit (Invent Biotechnologies, Plymouth, MN, USA) according to the manufacturer’s instructions. The collected membrane proteins were analyzed by western blotting.

### Western Blotting

Collected surface proteins were suspended in RIPA buffer (50 mM Tris-HCl, pH 7.4, 150 mM NaCl, 1 mM EDTA, 0.1% NP-40, 0.5% sodium deoxycholate, 0.1% sodium dodecyl sulfate), separated by sodium dodecyl sulfate-polyacrylamide gel electrophoresis, and then transferred electrophoretically onto polyvinylidene fluoride membranes (Millipore). After blocking with 2% BSA in TBS buffer (50 mM Tris-HCl, pH 7.4, 150 mM NaCl), the membranes were incubated with an anti-PAC1 antibody (1:1,000 dilution), anti-5-HT_1A_ antibody (1:1,000 dilution), anti-5-HT_2A_ antibody (1:1,000 dilution), anti-D2 antibody (1:1,000 dilution), anti-mGlu2/3 antibody (1:1,000 dilution), anti-β-actin antibody (1:2000 dilution) or anti-alpha 1 sodium potassium ATPase antibody (1:1000 dilution) overnight at 4°C. After incubation with a horseradish peroxidase-conjugated anti-rabbit IgG (1:2,000 dilution) or anti-mouse IgG (1:2,000 dilution) secondary antibody for 1 h at room temperature, proteins were detected by chemiluminescence and visualized with an ImageQuant LAS 4000 system (GE Healthcare, Little Chalfont, UK). For quantification, the bands of specific immune-complexes were analyzed using ImageJ software.

### Head Twitch Response and Intracerebroventricular Injections

Intracerebroventricular injections were performed as described previously (26). Head twitch responses were assessed as described previously (10). ICR mice were anesthetized and placed in a stereotaxic instrument (Narishige, Tokyo, Japan). A G-4 cannula (Eicom, Kyoto, Japan) was implanted, −0.4 mm posterior, 1.0 mm lateral, and 2.3 mm ventral from the bregma. After cannula implantation, each mouse was given 1 mg/kg buprenorphine (Sigma-Aldrich) to relieve pain and housed individually for at least 10 days before performing head-twitch experiments. Thirty minutes before DOI (Sigma-Aldrich) treatment, PACAP (10 pmol) was diluted in Ringer’s solution (1:100, Fuso Pharmaceutical Industries, Osaka, Japan) and a 3 μl volume was injected at an infusion rate of 1 μl/min using a microinjection pump (KD Scientific, MA, USA). For the pretreatment of the PAC1 antagonist, PACAP_6-38_ (100 pmol) were diluted and injected in the same way 30 min before PACAP treatment. The mice were individually placed in observation cages (19 × 10 × 11 cm) for a 30 min habituation period. They were then intraperitoneally injected with either saline or DOI, which were prepared just before use, and recordings were made for a duration of 60 min. Scoring began immediately after injection by trained observers who were blind to the treatment. The head twitch response is a distinctive paroxysmal head-twitching behavior that is easily distinguished from head-bobbing, lateral movements of the head and grooming. The intracerebroventricular injection was judged successful if the third ventricle was stained by Evans blue.

### Statistical Analysis

Experimental data were analyzed using Student’s *t*-test, or one-way, two-way or two-way repeated measures analysis of variance (ANOVA). The Tukey-Kramer *post hoc* test was also performed after significant main effects for interaction were observed. The criterion for statistical significance was *p* < 0.05. Statistical analyses were performed using StatView software (version 5.0; SAS Institute, Cary, NC, USA). All experiments were performed in a blinded manner. The observers were blinded to the group of samples during the analyses by random numbering.

## Results

### PACAP-Induced Internalization of 5-HT_2A_ in HEK293T Cells

To examine whether PACAP signaling modulates the internalization of 5-HT_2A_ and related GPCRs in HEK293T cells, we constructed membrane-specific Halo-tagged receptors for PAC1, 5-HT_2A_, 5-HT_1A_, 5-HT_2c_, D2 and mGlu2. As a first step, we examined whether *PAC1*, *VPAC1*, *VPAC2*, and *5-HT_2A_* mRNAs were expressed in HEK293T cells using reverse transcription (RT)-PCR analysis. In our HEK293T cell cultures, we detected the mRNA expression of *PAC1* and *VPAC1*; however, the expression of *VPAC2* and *5-HT_2A_* was below the detection limit of our RT-PCR analysis (**Supplementary Figure 1A**). Quantitative RT-PCR analysis showed that PC12 cells and SH-SY5Y cells expressed relatively higher levels of *PAC1* mRNA as expected from the previous reports (27–29), and both our HEK293T cell cultures and the HEK293T cells provided by RIKEN BRC Cell Bank (RCB2202; the National Bio-Resource Project of the MEXT/AMED, Japan) moderately expressed *PAC1* mRNA at similar levels. In Hela cells, *PAC1* expression was below the detection limit of our quantitative RT-PCR analysis (**Supplementary Figure 1B**). The nucleotide sequence of the cDNA fragment amplified from our HEK293T cell cultures was identical to that of the cDNA encoding the human PAC1 hop1 splice variant (NCBI Reference Sequence: NM_001199635.2).

We then examined whether PACAP, maxadilan, a potent and specific PAC1 agonist (30), and VIP increase intracellular cyclic adenosine monophosphate (cAMP) levels in our HEK293T cell cultures and confirmed that PACAP and maxadilan, both at ≥ 0.01 nM, significantly increased intracellular cAMP levels, while VIP at higher concentrations (≥ 1 nM) increased intracellular cAMP levels (**Supplementary Figure 1C**).

**Figure 1:**
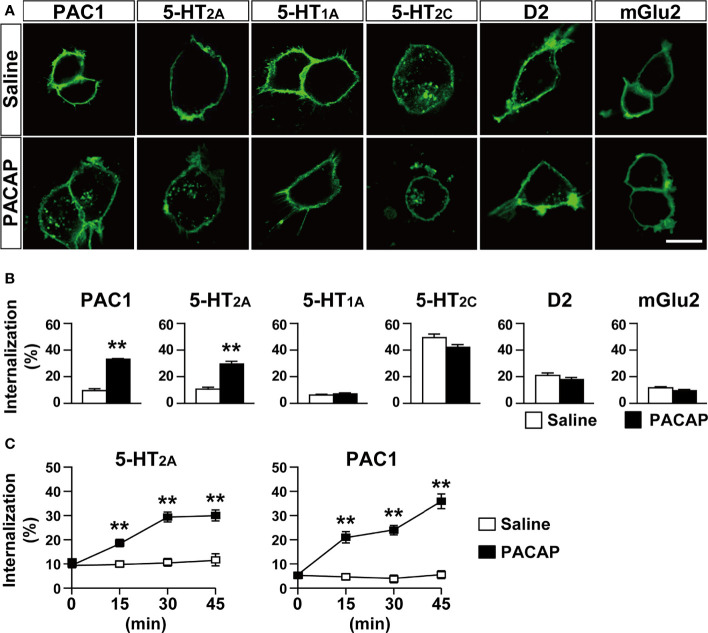
PACAP induces internalization of 5-HT_2A_ in HEK293T cells. **(A)** Representative images of HEK293T cells transfected with the indicated HaloTag receptors. The cells were labeled with Alexa Fluor 488 HaloTag membrane impermeable ligand for 15 min and then treated with 1 μM PACAP or saline for 30 min. Scale bar, 10 μm. **(B)** Quantification of the indicated HaloTag receptor internalization. Values are the mean ± SEM of 40–64 cells obtained from three independent experiments. **p < 0.01 vs. saline, Student’s *t*-test. **(C)** Time course of 5-HT_2A_ and PAC1 internalization for 45 min after PACAP treatment. Values are the mean ± SEM of 23–69 cells obtained from three independent experiments. **p < 0.01 vs. saline, two-way repeated-measures ANOVA followed by the Tukey-Kramer test.

To detect receptor internalization, only cell surface GPCR-Halo proteins were labeled with the cell-impermeable Alexa Fluor 488 HaloTag ligand and the signal ratio of internalized GPCR vs. total GPCR was determined in each cell after 30 min of PACAP treatment. PACAP (1 µM) induced an increase in the internalization of 5-HT_2A_ (saline, 10.64 ± 1.40; PACAP, 29.50 ± 2.07, *p* < 0.001, Student’s *t*-test) in HEK293T cells (**Figures 1A, B**). In accordance with previous reports (3, 4, 31, 32), PACAP also induced the internalization of PAC1 (saline, 9.52 ± 1.45; PACAP, 33.08 ± 0.56, *p* < 0.001, Student’s *t*-test) (**Figures 1A, B**). In contrast, PACAP did not affect the internalization of 5-HT_1A_ (saline, 6.19 ± 0.61; PACAP, 7.18 ± 0.64, not significant), 5-HT_2c_ (saline, 49.35 ± 2.72; PACAP, 42.06 ± 2.09, not significant), D2 (saline, 20.95 ± 1.93; PACAP, 17.87 ± 1.81, not significant), or mGlu2 (saline, 11.64 ± 0.84; PACAP, 9.44 ± 0.95, not significant) (**Figures 1A, B**). We also analyzed the time course of PACAP-induced internalization. The internalization ratios of 5-HT_2A_ and PAC1 were similarly increased within 15 min after PACAP treatment and remained elevated for at least 45 min (two-way repeated-measures ANOVA; 5-HT_2A_, treatment effect, *F*_(1, 82)_ = 65.77, *p* < 0.001; time effect, *F*_(3, 246)_ = 11.77, *p* < 0.001; interaction, *F*_(3, 246)_ = 11.75, *p* < 0.001; PAC1, treatment effect, *F*_(1, 54)_ = 96.14, *p* < 0.001; time effect, *F*_(3, 162)_ = 19.41, *p* < 0.001; interaction, *F*_(3, 162)_ = 18.66, *p* < 0.001) (**Figure 1C**). In accordance with previous reports (15, 16), 5-HT increased 5-HT_2A_ internalization in a time-dependent manner, the pattern of which was similar to that of PACAP-induced 5-HT_2A_ internalization (**Supplementary Figure 2**).

### PAC1 Mediates PACAP-Induced 5-HT_2A_ Internalization

**Figure 2:**
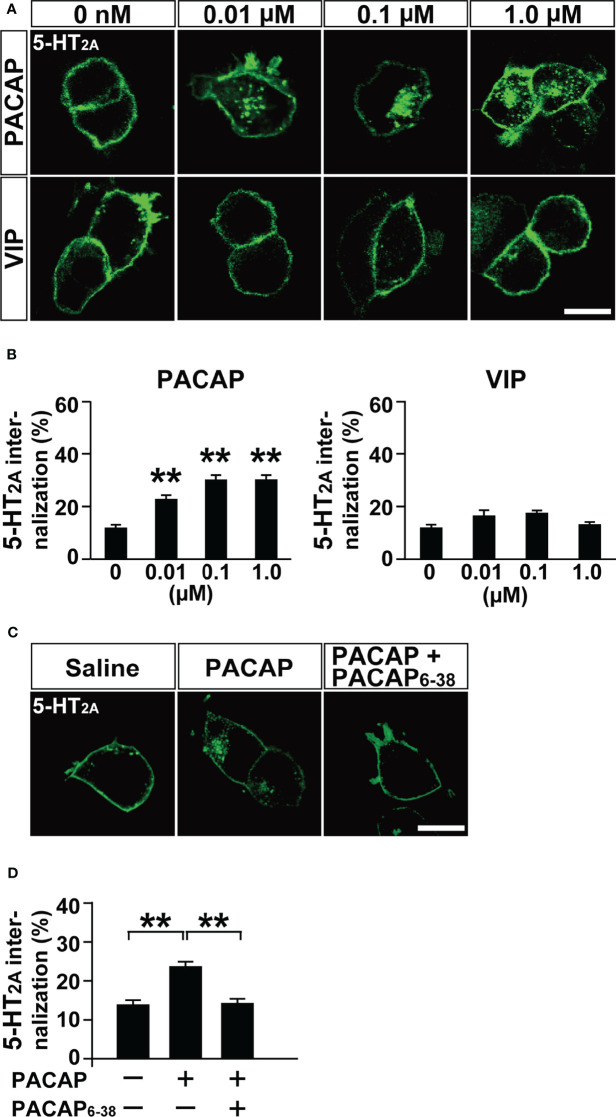
PACAP induces 5-HT_2A_ internalization *via* PAC1 in HEK293T cells. **(A)** Representative images of HEK293T cells transfected with HaloTag 5-HT_2A_. The cells were labeled with Alexa Fluor 488 HaloTag membrane impermeable ligand for 15 min and then treated with the indicated concentrations of PACAP or VIP for 30 min. Scale bar, 10 μm. **(B)** Quantification of 5-HT_2A_ internalization. Values are the mean ± SEM of 46–71 cells obtained from three independent experiments. **p < 0.01 vs. 0 μM, one-way ANOVA followed by the Tukey-Kramer test. **(C)** Representative images of HEK293T cells transfected with 5-HT_2A_. The cells were pretreated with 2 μM PACAP_6-38_ or saline for 30 min, labeled with Alexa Fluor 488 HaloTag membrane impermeable ligand for 15 min and then treated with 100 nM PACAP or saline for 30 min. Scale bar, 10 μm. **(D)** Quantification of 5-HT_2A_ internalization. Values are the mean ± SEM of 80–86 cells obtained from three independent experiments. **p < 0.01, one-way ANOVA followed by the Tukey-Kramer test.

To examine the subtypes of the three PACAP receptors (PAC1, VPAC1 and VPAC2) involved in PACAP-induced 5-HT_2A_ internalization, we compared 5-HT_2A_ internalization following administration of various doses of PACAP and VIP. PACAP (0.01, 0.1, and 1 µM) dose-dependently increased 5-HT_2A_ internalization (one-way ANOVA, *F*_(3, 321)_ = 29.44, *p* < 0.001), but VIP (0.01, 0.1, and 1 µM) did not (one-way ANOVA, *F*_(3, 304)_ = 3.62, *p* = 0.054) (**Figures 2A, B**). Pretreatment with PACAP_6-38_, a PAC1 antagonist, significantly inhibited the PACAP-induced 5-HT_2A_ internalization (one-way ANOVA, *F*_(2, 246)_ = 17.54, *p* < 0.001) (**Figures 2C, D**). In addition, shRNA-mediated PAC1 silencing in HEK293T cells, which effectively decreased *PAC1* mRNA levels to less than 5% of normal levels, blocked the PACAP-induced 5-HT_2A_ internalization (two-way ANOVA, PACAP effect, *F*_(1, 156)_ = 79.51, *p* < 0.001; shRNA effect, *F*_(1, 156)_ = 76.58, *p* < 0.001; interaction, *F*_(1, 156)_ = 81.72, *p* < 0.001) (**Supplementary Figure 3**). Taken together these results indicate that PAC1 is involved in PACAP-induced 5-HT_2A_ internalization.

### PKC Is Involved in PACAP-Induced 5-HT_2A_ Internalization

**Figure 3:**
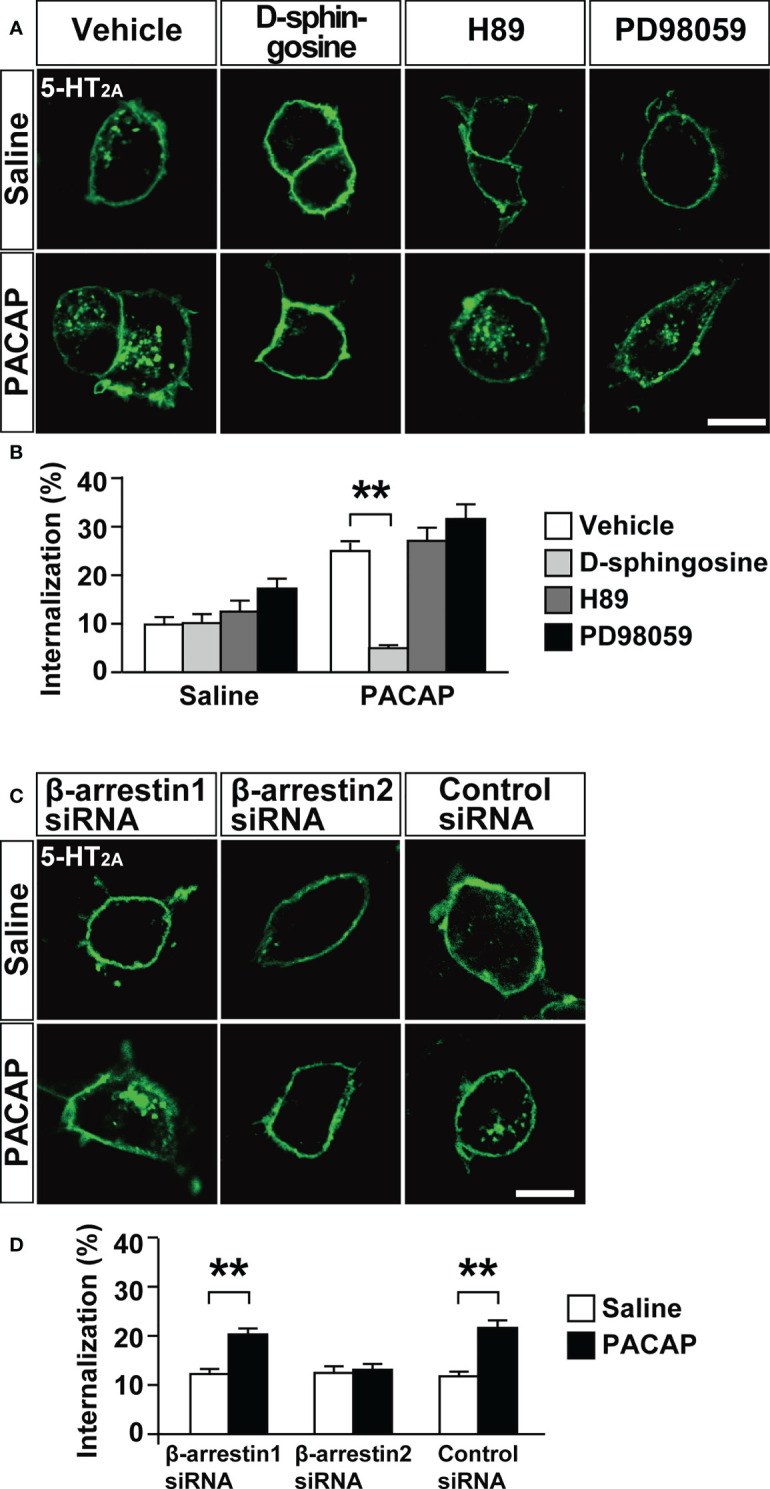
Effect of kinase inhibitors or β-arrestin silencing on PACAP-induced 5-HT_2A_ internalization in HEK293T cells. **(A)** Representative images of HEK293T cells transfected with 5-HT_2A_. The cells were pretreated with 50 μM D-sphingosine (PKC inhibitor), 20 μM H89 (protein kinase A inhibitor), 50 μM PD98059 (MEK inhibitor) or saline for 30 min, labeled with Alexa Fluor 488 HaloTag membrane impermeable ligand for 15 min and then treated with 1 μM PACAP or saline for 30 min. Scale bar, 10 μm. **(B)** Quantification of 5-HT_2A_ internalization. Values are the mean ± SEM of 34–51 cells obtained from three independent experiments. **p < 0.01, two-way ANOVA followed by the Tukey-Kramer test. **(C)** Representative images of HEK293T cells cotransfected with 5-HT_2A_ plus β-arrestin1 siRNA, β-arrestin2 siRNA or the negative control siRNA. The cells were labeled with Alexa Fluor 488 HaloTag membrane impermeable ligand for 15 min and then treated with 1 μM PACAP or saline for 30 min. Scale bar, 10 μm. **(D)** Quantification of 5-HT_2A_ internalization. Values are the mean ± SEM of 77–87 cells obtained from three independent experiments. **p < 0.01, two-way ANOVA followed by the Tukey-Kramer test.

We then addressed the signaling pathways involved in PACAP-induced 5-HT_2A_ internalization. Pretreatment with the PKC inhibitor D-sphingosine (50 µM), but not the protein kinase A inhibitor H89 (20 µM), or the mitogen-activated protein kinase kinase (MEK) inhibitor PD98059 (50 µM), blocked the PACAP-induced 5-HT_2A_ internalization (two-way ANOVA, PACAP effect, *F*_(1, 372)_ = 44.34, *p* < 0.001; inhibitor effect, *F*_(3, 372)_ = 18.41, *p* < 0.001; interaction, *F*_(3, 372)_ = 8.04, *p* < 0.001) (**Figures 3A, B**). Another PKC inhibitor 1-(5-isoquinolinesulfonyl)-2-methylpiperazine dihydrochloride (H7) also significantly blocked PACAP-induced 5-HT_2A_ internalization, whereas HA1004, a structural analog of H7 and used as a control, did not significantly inhibit the PACAP-induced 5-HT_2A_ internalization (two-way ANOVA, PACAP effect, *F*_(1, 448)_ = 33.37, *p* < 0.001; inhibitor effect, *F*_(2, 448)_ = 8.98, *p* < 0.001; interaction, *F*_(2, 448)_ = 4.53, *p* = 0.011) (**Supplementary Figure 4**).

### β-Arrestin2 Is Involved in PACAP-Induced 5-HT_2A_ Internalization

We recently reported that β-arrestin2, but not β-arrestin1, is involved in PACAP-induced internalization of PAC1 (4). We therefore examined whether β-arrestins are also involved in PACAP-induced 5-HT_2A_ internalization. Although the β-arrestin1 and β-arrestin2 siRNAs effectively decreased respective β-arrestin levels to less than 35% of normal levels in HEK293T cells (4), β-arrestin2 siRNA, but not β-arrestin1 siRNA, blocked the PACAP-induced 5-HT_2A_ internalization (two-way ANOVA, PACAP effect, *F*_(1, 490)_ = 37.78, *p* < 0.001; silencing effect, *F*_(2, 490)_ = 5.85, *p* = 0.0031; interaction, *F*_(2, 490)_ = 7.61, *p* < 0.001) (**Figures 3C, D**). A negative control siRNA showed no effect on PACAP-induced 5-HT_2A_ internalization (**Figures 3C, D**).

### PACAP Decreases Cell Surface Localization of Endogenously Expressed 5-HT_2A_

**Figure 4:**
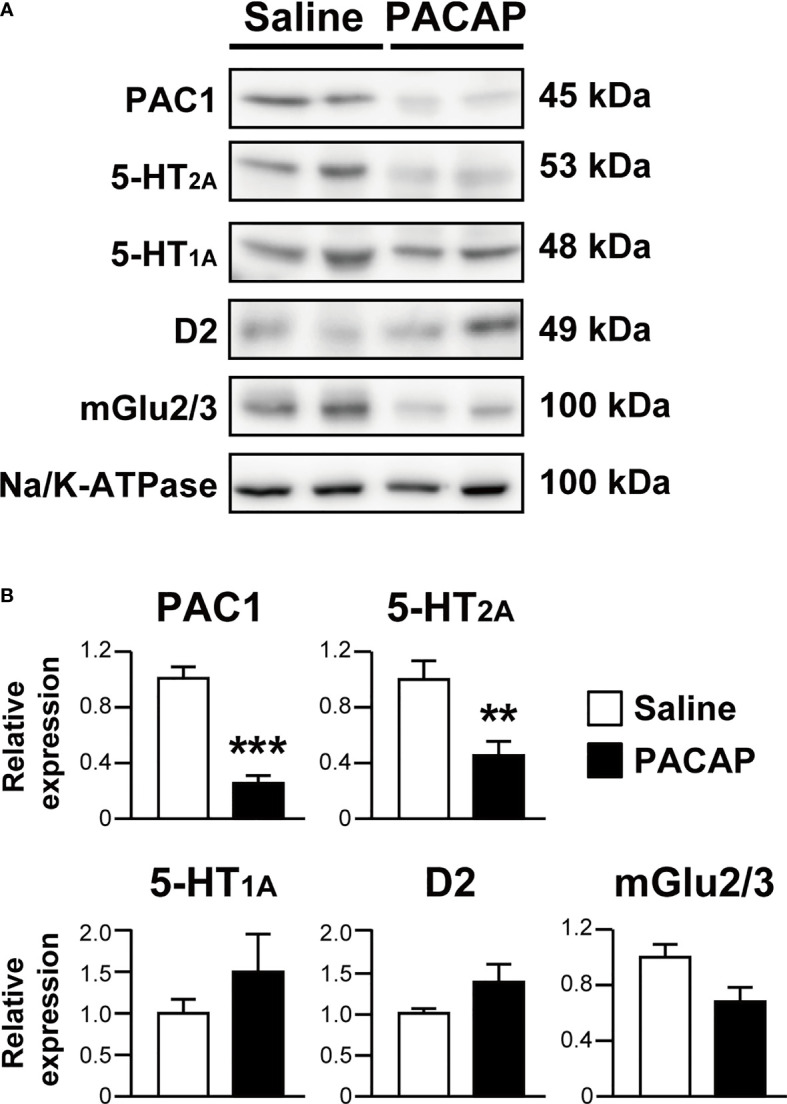
PACAP significantly decreases cell surface localization of 5-HT_2A_ in primary cultured cortical neurons. **(A)** Representative immunoblots of cell surface biotinylated PAC1, 5-HT_2A_, 5-HT_1A_, D2, mGlu2/3 and alpha 1 sodium potassium ATPase (Na/K-ATPase) in primary cultured cortical neurons at 14 days *in vitro* treated with 1 μM PACAP or saline for 30 min. The band size is indicated for each blot. **(B)** Quantification of cell surface levels of PAC1, 5-HT_2A_, 5-HT_1A_, D2 and mGlu2/3 normalized to the levels of Na/K-ATPase. Values are the mean ± SEM from three or four independent experiments. **p < 0.01, ***p < 0.001 vs. saline, Student’s *t*-test.

To confirm the phenomenon of PACAP-induced 5-HT_2A_ internalization in more neurologically relevant cells, we examined the effect of PACAP on the cell surface localization of endogenously expressed 5-HT_2A_ in mouse primary cultured cortical neurons using a cell surface biotinylation assay. PACAP significantly decreased the levels of cell-surface biotinylated 5-HT_2A_ (saline, 1.00 ± 0.14; PACAP, 0.46 ± 0.10; *p* = 0.0077, Student’s *t*-test) (**Figures 4A, B**). As expected, cell-surface biotinylated PAC1 levels were also decreased by PACAP (saline, 1.00 ± 0.09; PACAP, 0.26 ± 0.055; *p* < 0.001, Student’s *t*-test) (**Figures 4A, B**). In contrast, levels of cell-surface biotinylated 5-HT_1A_ (saline, 1.00 ± 0.17; PACAP, 1.50 ± 0.45; not significant), D2 (saline, 1.00 ± 0.058; PACAP, 1.37 ± 0.22; not significant) and mGlu2/3 (saline, 1.00 ± 0.10; PACAP, 0.68 ± 0.13; not significant) were not affected by PACAP (**Figures 4A, B**).

**Figure 5:**
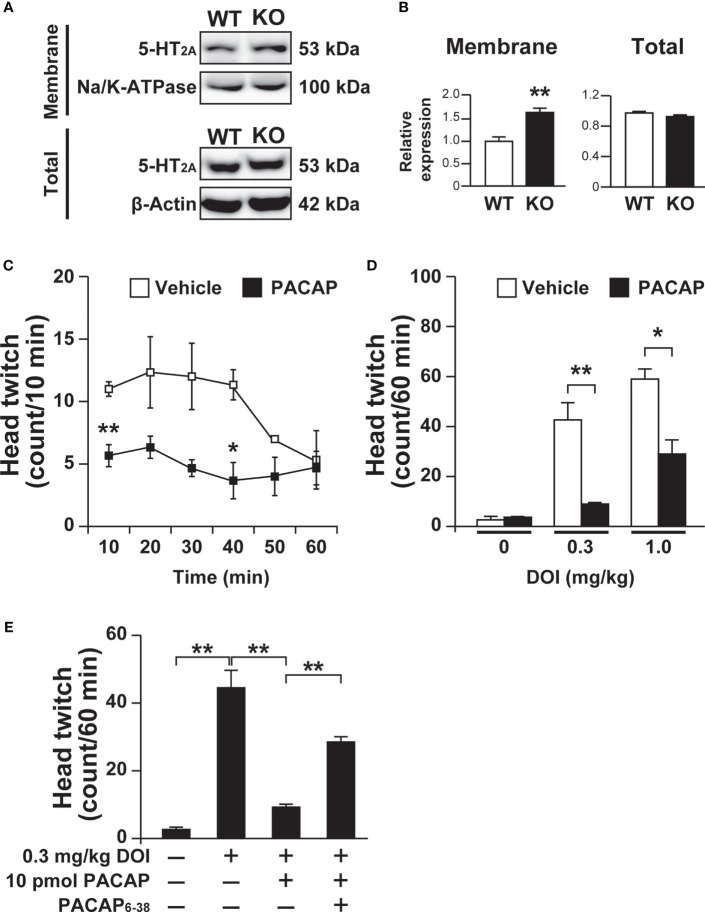
Increased 5-HT_2A_ levels in the membrane fraction of the frontal cortex in *Pacap^–/–^* mice and PACAP-induced attenuation of DOI-induced head twitch response. **(A)** Representative immunoblots of 5-HT_2A_ in the cell membrane fraction (membrane) or total cell lysate (total) of the frontal cortex from wild-type (WT) or *Pacap^–/–^* (KO) mice. As internal controls, Na/K-ATPase (membrane) and β-actin (total) were used. **(B)** Quantification of 5-HT_2A_ levels normalized to Na/K-ATPase (membrane) or β-actin (total). Values are the mean ± SEM (n = 5). **p < 0.01 vs. saline, Student’s *t*-test. **(C, D)** Mice intracerebroventricularly administered PACAP (10 pmol) or vehicle were treated with DOI and their head-twitch responses were counted. **(C)** Time course of DOI (1 mg/kg)-induced head twitch responses. **(D)** Head twitch responses during 60 min in mice injected with the indicated doses of DOI. Values are the mean ± SEM (n = 3 per group). *p < 0.05, **p < 0.01 vs. vehicle, two-way repeated measures ANOVA **(C)** and two-way ANOVA **(D)** followed by the Tukey-Kramer test. **(E)** Effect of the PAC1 antagonist PACAP_6-38_ on the PACAP inhibition of DOI-induced head twitch response. Thirty minutes before PACAP administration, PACAP_6-38_ (100 pmol) were preadministered intracerebroventricularly. Values are the mean ± SEM (n = 4 per group). **p < 0.01 vs. vehicle, one-way ANOVA followed by the Tukey-Kramer test.

In addition, 5-HT_2A_ levels in the membrane fraction of the frontal cortex were increased in *Pacap^–/–^* mice compared with wild-type mice (saline, 1.00 ± 0.10; PACAP, 1.64 ± 0.10; *p* = 0.002, Student’s *t*-test), although no significant change was observed in total 5-HT_2A_ protein levels between *Pacap^–/–^* and wild-type mice (saline, 1.00 ± 0.03; PACAP, 0.94 ± 0.03; not significant, Student’s *t*-test) (**Figures 5A, B**).

### Intracerebroventricular PACAP Administration Ameliorates the Hallucinogenic Head Twitch Response

We then addressed PACAP signaling involvement in 5-HT_2A_-dependent behavioral responses by examining the head twitch response, which is a characteristic head-shaking movement induced by a hallucinogenic drug through the stimulation of 5-HT_2_ receptors (33). DOI (1.0 mg/kg)-induced head twitch responses were significantly fewer in mice administered PACAP (10 pmol) compared with vehicle control mice in the first, third and fourth 10 min-bins of a 60-min observation period (**Figure 5C**). The numbers of head twitch responses induced by 0.3 and 1.0 mg/kg DOI during 60 min were significantly lower in mice administered PACAP compared with vehicle control mice (two-way ANOVA, PACAP effect, *F*_(1, 12)_ = 39.80, *p* < 0.001; dose effect, *F*_(2, 12)_ = 50.90, *p* < 0.001; interaction, *F*_(2, 12)_ = 11.03, *p* = 0.0019) (**Figure 5D**). In addition, we examined whether the inhibitory effect of PACAP on DOI-induced head twitch response is mediated by PAC1 by using the PAC1 antagonist PACAP_6-38_. Intracerebroventricular preadministration of PACAP_6-38_ (100 pmol) significantly blocked the inhibitory effect of PACAP on DOI-induced head twitch response (one-way ANOVA, *F*_(3, 12)_ = 47.77, *p* < 0.001) (**Figure 5E**).

## Discussion

In the present study, we investigated the mechanisms underlying the relationship between PACAP and 5-HT_2A_ signaling pathways. We found that PACAP time- and dose-dependently increased the internalization of 5-HT_2A_, but not 5-HT_1A_, 5-HT_2c_, D2 or mGlu2, in HEK293T cells and that the effect of PACAP was mediated by PAC1, PKC and β-arrestin2. In addition, we showed that PACAP decreased the cell surface levels of endogenously expressed 5-HT_2A_ in mouse primary cultured cortical neurons and that 5-HT_2A_ levels in the membrane fraction of the frontal cortex were increased in *Pacap^–/–^* mice compared with wild-type mice. Finally, we observed that intracerebroventricular administration of PACAP suppressed DOI-induced head twitch responses in mice. These results suggest that PACAP–PAC1 signaling increases 5-HT_2A_ internalization, resulting in attenuation of 5-HT_2A_-meadiated signaling.

In the present study, it is still uncertain whether PACAP-induced 5-HT_2A_ internalization can be a mechanism for behavioral abnormalities including hyperactivity, PPI deficits, depressive-like behavior and memory impairment, reversal of the depressive-like behavior by the 5-HT_2A_ antagonist ritanserin, and exaggerated DOI-induced hallucinogenic behaviors in *Pacap^–/–^* mice. In order to address this, it is necessary to examine if increased cell surface expression of 5-HT_2A_ in the frontal cortex (and possibly other brain regions as well) is relevant to behavioral impairments including exaggerated DOI-induced hallucinogenic behaviors and the effects of 5-HT_2A_ antagonists on reversal of the impairments in *Pacap* ^–/–^ mice (5–10). Given that increased cell surface expression of 5-HT_2A_ leads to supersensitivity of the 5-HT_2A_-mediated 5-HT response, it is reasonable that 5-HT_2A_ antagonists effectively reverse the behavioral impairments in *Pacap* ^–/–^ mice. The issue should also be addressed by examining whether 5-HT_2A_ antagonists affect PAC1 and 5-HT_2A_ interactions.

We examined 5-HT_2A_ levels in the membrane fraction of the frontal cortex in *Pacap^–/–^* mice, since both 5-HT_2A_, PACAP and PAC1 are expressed in this brain region (34–37), suggesting a potential colocalization of 5-HT_2A_ and PAC1 in the frontal cortex. In addition, 5-HT_2A_ expressed in the frontal cortex plays an important role in the pathophysiology and therapeutic effects of schizophrenia (12, 14). However, further analyses in other brain regions are needed, which will be investigated in our future work.

5-HT_2A_ internalization is involved in diverse signaling pathways depending on different ligands. Recent studies indicate that 5-HT_2A_ internalization signaling may be separated into hallucinogenic and antipsychotic specific pathways, because hallucinogenic and non-hallucinogenic 5-HT_2A_ ligands induce distinct immediate early gene expression patterns (38–41). Hallucinogenic DOI-induced 5-HT_2A_ internalization is independent on β-arrestins and antipsychotic clozapine-mediated internalization is independent on PKC (16, 42). Urs et al. (43) reported that β-arrestin-biased D2 ligands exert unique brain region-specific antipsychotic actions (43). The present observation that PACAP–PAC1 signaling regulates 5-HT_2A_ internalization in a PKC- and β-arrestin2-dependent manner provides a new molecular mechanism for this peptidergic signaling that cross-talks with serotonergic signaling in the brain.

We also examined the protein-protein interaction between PAC1 and 5-HT_2A_ by co-immunoprecipitation using an anti-5-HT_2A_ antibody; however, co-immunoprecipitation of PAC1 with 5-HT_2A_ was not detected (data not shown). Therefore, it remains unclear how PACAP–PAC1 signaling induces 5-HT_2A_ receptor internalization. We previously reported that PACAP–PAC1 signaling markedly reduces the association between DISC1 and DBZ in PC12 cells (44). DISC1 forms a protein complex of DISC1/Kalirin-7/PSD-95 (45). The Kalirin-7/PSD-95 complex is also directly associated with the 5-HT_2A_ receptor and regulates 5-HT_2A_ signaling and trafficking in HEK293 cells (46, 47). In addition, we previously showed that β-arrestin2, but not β-arrestin1, was involved in PACAP-induced internalization of PAC1 (4). PACAP–PAC1 signaling may regulate 5-HT_2A_ internalization through these adaptor proteins.

In the present study, we observed, in our HEK293T cell cultures, expression of PAC1 transcript, maxadilan-induced cAMP elevation, PACAP-induced 5-HT_2A_ internalization as well as inhibition of the PACAP-induced 5-HT_2A_ internalization by PACAP_6-38_ and shRNA-mediated PAC1 silencing. In addition, we observed that the HEK293T cells which was newly obtained from RIKEN BRC Cell Bank expressed *PAC1* mRNA at a similar level with our HEK293T cell cultures used in the present 5-HT_2A_ internalization study. However, previous studies have shown that HEK293T cells did not express PAC1 (3, 28, 48, 49) and therefore PAC1 was exogenously expressed to investigate the signal transduction system. In contrast, it was also reported that HEK293T cells expressed the PAC1 protein as observed by western blot analysis (50, 51). The reason for the disagreement in PAC1 expression in HEK293T cells is currently unknown but might be related with passage number and culture conditions.

Serotonin syndrome is caused by adverse side effects of serotonergic drugs and is associated with increased serotoninergic activity (52). By indirectly antagonizing 5-HT_2A_ function, PACAP signaling may have the potential to ameliorate serotonin syndrome. Accumulating evidence suggests that PACAP–PAC1 signaling in the brain provides clues to elucidating the pathomechanisms of neurological and psychiatric disorders (53–55). The present study furthers understanding of PACAP–PAC1 signaling and shows that this pathway is a promising target for the development of neurotherapeutics.

## Data Availability Statement

The raw data supporting the conclusions of this article will be made available by the authors.

## Ethics Statement

This animal study was reviewed and approved by the Animal Care and Use Committee of the Graduate School of Pharmaceutical Sciences, Osaka University.

## Author Contributions

AH-T: design, experimentation, statistics, visualization, and writing. YS: experimentation and statistics. KM: experimentation and statistics. NE: experimentation and statistics. KK: experimentation. TN: writing and supervision. HH: conception, writing, and supervision. All authors contributed to the article and approved the submitted version.

## Funding

This work was supported in part by the Japan Society for the Promotion of Science (JSPS) KAKENHI, grant numbers JP16K08269 (AH-T), JP19K07121 (AH-T), JP20H00492 (HH), JP20H03429 (HH, AH-T), JP20K07736 (HH, AH-T), JP21K19335 (HH), MEXT KAKENHI, grant number JP18H05416 (HH), AMED, grant numbers JP21dm0207117 (HH), and JP21am0101084 (HH), and a grant from the Takeda Science Foundation (HH).

## Conflict of Interest

The authors declare that the research was conducted in the absence of any commercial or financial relationships that could be construed as a potential conflict of interest.

## Publisher’s Note

All claims expressed in this article are solely those of the authors and do not necessarily represent those of their affiliated organizations, or those of the publisher, the editors and the reviewers. Any product that may be evaluated in this article, or claim that may be made by its manufacturer, is not guaranteed or endorsed by the publisher.
