# Supplementary material for: PACAP–PAC1 Signaling Regulates Serotonin 2A Receptor Internalization
Source: Front Endocrinol (Lausanne). 2021 Oct 25;12:732456. doi: 10.3389/fendo.2021.732456 (PMC8574227; doi:10.3389/fendo.2021.732456)
Supplement: Supplementary file 1 [file DataSheet_1.pdf]

Supplementary Information

PACAP-PAC1 signaling regulates serotonin 2A receptor internalization

Atsuko Hayata-Takano, Yusuke Shintani, Keita Moriguchi, Naoki Encho, Kohei Kitagawa, Takanobu Nakazawa, Hitoshi Hashimoto

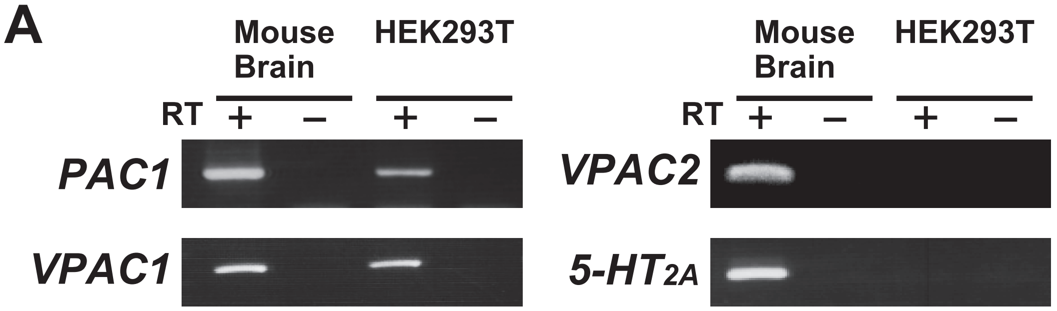

B

Quantitative RT-PCR threshold cycle number

	HEK293T our cultures	HEK293T RIKEN BRC	PC12	SH-SY5Y	Hela
Average	30.67	29.53	24.71	24.43	n.d.
mean ± SEM	0.61	1.12	0.16	0.34	–

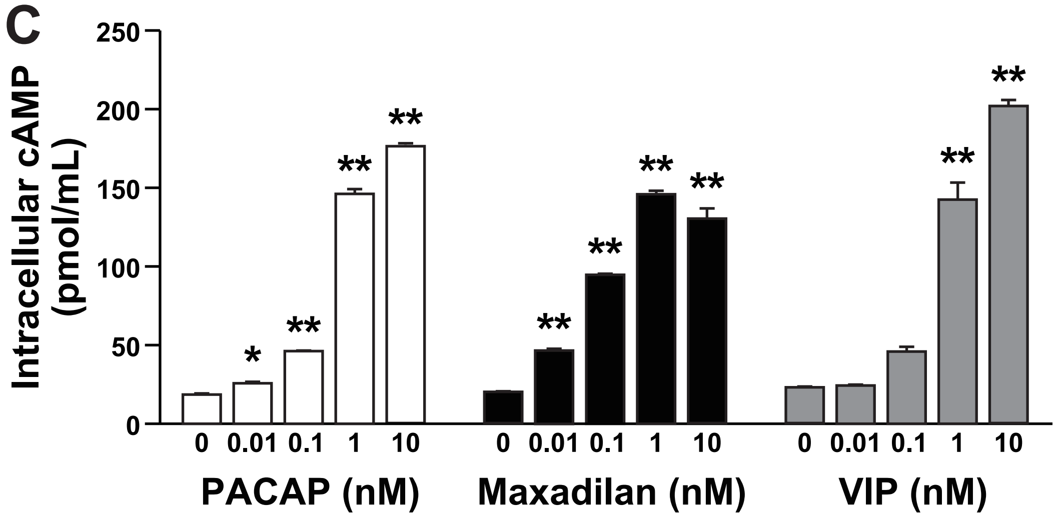

Supplementary Fig. 1. PAC1 expression in HEK293T cells

(A) Reverse transcription (RT)-PCR analysis of mRNA levels of three PACAP receptor subtypes and 5-HT_{2A} in our HEK293T cell cultures. PCR was performed on cDNAs generated from HEK293T cells and mouse brain (positive control). +, RT-positive , –, RT-negative. (B) Quantitative RT-PCR analysis for *PAC1* mRNA expression in our HEK293T cell cultures, the HEK293T cells provided by RIKEN BRC Cell Bank, PC12 cells, SH-SY5Y cells, and Hela cells. Values represent threshold cycle numbers and are the mean ± SEM (n = 3). n.d., not detected. (C) PACAP, maxadilan and VIP-induced increase in intracellular cAMP levels in HEK293T cells (our cultures). HEK293T cells were treated with the indicated concentrations of PACAP, maxadilan and VIP for 20 min, and cAMP levels were determined. Values are the mean ± SEM (n = 3). *$p < 0.05$, **$p < 0.01$ vs. 0 nM, one-way ANOVA followed by Tukey-Kramer test.

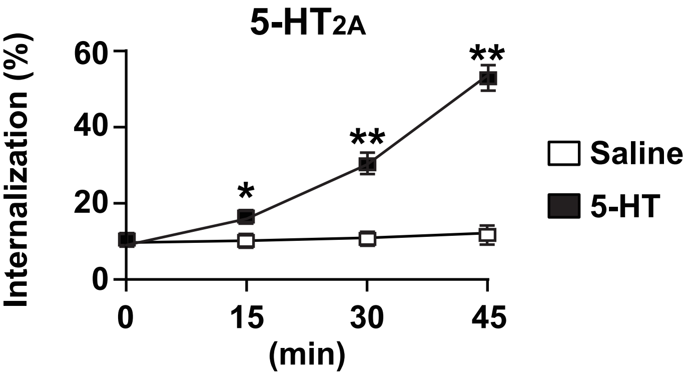

Supplementary Fig. 2. Time course of serotonin-induced 5-HT$_{2A}$ internalization
Time course of 5-HT$_{2A}$ internalization for 45 min after serotonin or saline treatment. Values are the mean ± SEM of 37–69 cells obtained from three independent experiments. **p* < 0.05, ***p* < 0.01 vs. saline, two-way repeated-measures ANOVA followed by Tukey-Kramer test.

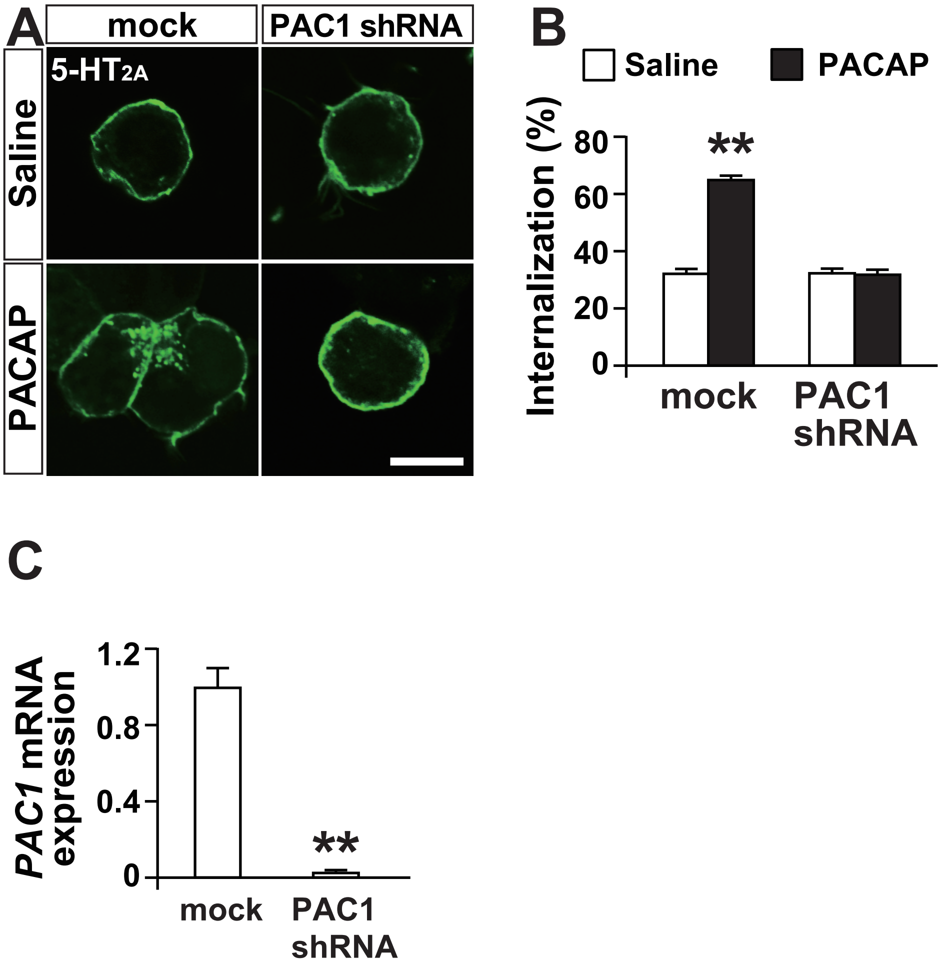

Supplementary Fig. 3. Effect of PAC1 silencing on PACAP-induced 5-HT_{2A} internalization in HEK293T cells

(A) Representative images of HEK293T cells cotransfected with *5-HT_{2A}* plus PAC1 shRNA or mock. The cells were labeled with Alexa Fluor 488 HaloTag membrane impermeable ligand for 15 min and then treated with 1 μM PACAP or saline for 30 min. Scale bar, 10 μm. (B) Quantification of 5-HT_{2A} internalization. Values are the mean ± SEM of 40 cells obtained from three independent experiments. ***p* < 0.01 vs. saline, two-way ANOVA followed by Tukey-Kramer test.(C) *PAC1* mRNA levels in HEK293T cells transfected with PAC1 shRNA. Values are the mean ± SEM from three independent experiments. ***p* < 0.01 vs. mock, one-way ANOVA followed by Tukey-Kramer test.

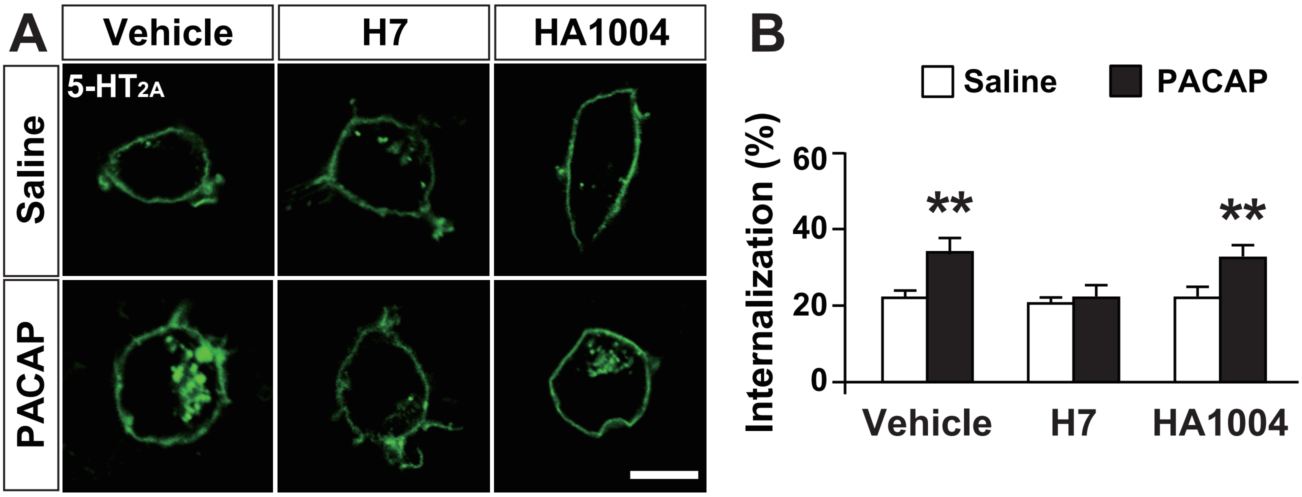

Supplementary Fig. 4. Effect of the PKC inhibitor, H7, and its structural analog, HA1004, on the PACAP-induced 5-HT$_{2A}$ internalization in HEK293T cells

(A) Representative images of HEK293T cells transfected with *5-HT$_{2A}$*. The cells were pretreated with 100 μM H7, 100 μM HA1004 or vehicle for 30 min, labeled with Alexa Fluor 488 HaloTag membrane impermeable ligand for 15 min and then treated with 1 μM PACAP or saline for 30 min. Scale bar, 10 μ m. (B) Quantification of 5-HT$_{2A}$ internalization. Values are the mean ± SEM of 70-80 cells obtained from three independent experiments. $^{**}p < 0.01$ vs. saline, two-way ANOVA followed by Tukey-Kramer test.

Table 1. Primers used for PCR analyses

Genes	Oligonucleotide_sequences (5'→3')	Size fragment (bp)	Annealing temperature (°C)	Genbank ID
**Human *PAC1***	AGCTGCACTGCACACGCAAC[1)]<br>GCTGTGCTGTCATTCATATCC[1)]	373	52	NM_001199635.2
**Human *PAC1***	CCATGTGGGTGAGATGGTCC[2)]<br>AGCAAGAGCAGCTCCCAAAT[2)]	1250	60	NM_001199635.2
**Human *5-HT2A***	CCGCTTCAACTCCAGAACTAAGGC<br>CTTCGAATCGTCCTGTAGCCCAAA	109	56	NM_001165947
**Human *VPAC1***	TGCAGCAAGATGTGGGACAA<br>GTCTGCTGCTCATCCAAACT	221	62	NM_001251885
**Human *VPAC2***	TGGGATACAAACGACCACAG<br>TGTACTGAGACTGGTCGTTGC	157	58	NM_003382
**Human *GAPDH***	CAACGACCACTTTGTCAAGC<br>GGTGGTCCAGGGGTCTTACT	115	60	NM_002046
**Mouse *PAC1***	AGCTGCACTGTACCCGTAAC[1)]<br>GCTGTGCTGTCATTCATATCC[1)]	373	52	NM_001315504
**Rat *PAC1***	TTGCAAGATGTCAGAACTATCCA<br>GAAGTAACGGTTCACCTTCCAG	259	60	NM_001270579

[1)]Primers used for RT-PCR and quantitative RT-PCR.

[2)]Primers used for RT-PCR followed by nucleotide sequencing.

Supplementary methods

PCR and qRT-PCR analysis

Total RNAs from HEK293T cells, PC12 cells, SH-SY5Y cells, Hela cells, and mouse brain tissue were extracted using the guanidine-isothiocyanate procedure. Reverse transcription of the total RNA (1 μg) was performed as previously described (1). The PCR was carried out using GoTaq G2 Hot Start Green Master Mix (Promega, Madison, WI, USA). The PCR consisted of 30 cycles each denaturation at 95 °C for 10 s, annealing at 52–62 °C for 20 s, and extension at 72 °C for 20 s. The quantitative RT-PCR was carried out using GoTaq qPCR Master Mix (Promega) and performed as previously described (2). The *GAPDH* housekeeping gene was simultaneously reverse transcribed and amplified as the internal reference for the experiment in Supplementary Figure 3C. The real-time PCR consisted of up to 40 cycles each denaturation at 95 °C for 10 s, annealing at 52 °C or 60 °C for 20 s, and extension at 72 °C for 20 s. The primer sequences and annealing temperatures of the PCR cycles are indicated in Table 1. The nucleotide sequence of the amplified fragment separated on agarose gel electrophoresis was determined by Eurofins Genomics (Tokyo, Japan).

cAMP assay

Intracellular cAMP levels in HEK293T cells were measured using the cAMP enzyme-linked immunosorbant assay (ELISA) kit (Cayman Chemicals, #501040, Grand Rapids, MI, USA) following the manufacturer’s instructions. HEK293T cells were plated in 6-well plates at a density of 1×10^6 cells per well and incubated for 24 h. After the cells were treated with PACAP, maxadilan (kindly provided by Dr. Atsuro Miyata at Kagoshima University) (3) or VIP for 20 min, cAMP levels were determined.

PAC1 silencing

For shRNA-induced silencing of PAC1, PAC1 MISSION shRNA was purchased from Sigma-Aldrich (Cat#SHCLNG-NM_001118). PAC1 shRNA lentiviral was prepared as described previously (2). The expression levels of *PAC1* mRNA were measured by quantitative RT-PCR

and normalized to those of *GAPDH* mRNA. The PAC1 shRNA effectively decreased the *PAC1* mRNA levels to less than 5% in HEK293T cells.

Supplementary references

1. Mabuchi T., Shintani N., Matsumura S., Okuda-Ashitaka E., Hashimoto H., Muratani T. et al. Pituitary adenylate cyclase-activating polypeptide is required for the development of spinal sensitization and induction of neuropathic pain. J Neurosci (2004) 24:7283-91. doi:10.1523/JNEUROSCI.0983-04.2004
2. Hayata-Takano A., Kamo T., Kijima H., Seiriki K., Ogata K., Ago Y. et al. Pituitary Adenylate Cyclase-Activating Polypeptide Modulates Dendritic Spine Maturation and Morphogenesis via MicroRNA-132 Upregulation. J Neurosci (2019) 39:4208-20. doi:10.1523/JNEUROSCI.2468-18.2019
3. Uchida D., Tatsuno I., Tanaka T., Hirai .A, Saito Y., Moro O. et al. Maxadilan is a specific agonist and its deleted peptide (M65) is a specific antagonist for PACAP type 1 receptor. Ann N Y Acad Sci (1998)865:253–258 doi:10.1111/j.1749-6632.1998.tb11185.x
